# Microvesicles in Atherosclerosis and Angiogenesis: From Bench to Bedside and Reverse

**DOI:** 10.3389/fcvm.2017.00077

**Published:** 2017-12-18

**Authors:** Lina Badimon, Rosa Suades, Gemma Arderiu, Esther Peña, Gemma Chiva-Blanch, Teresa Padró

**Affiliations:** ^1^Cardiovascular Research Center (ICCC) and CiberCV, Sant Pau Biomedical Research Institute (IIB-Sant Pau), Barcelona, Spain; ^2^Cardiovascular Research Chair, UAB, Barcelona, Spain

**Keywords:** angiogenesis, atherosclerosis, cardiovascular diseases, cell-derived microvesicles, endothelial dysfunction, inflammation, neovascularization, thrombosis

## Abstract

Atherosclerosis (AT) is a progressive chronic disease involving lipid accumulation, fibrosis, and inflammation in medium and large-sized arteries, and it is the main cause of cardiovascular disease (CVD). AT is caused by dyslipidemia and mediated by both innate and adaptive immune responses. Despite lipid-lowering drugs have shown to decrease the risk of cardiovascular events (CVEs), there is a significant burden of AT-related morbidity and mortality. Identification of subjects at increased risk for CVE as well as discovery of novel therapeutic targets for improved treatment strategies are still unmet clinical needs in CVD. Microvesicles (MVs), small extracellular plasma membrane particles shed by activated and apoptotic cells have been widely linked to the development of CVD. MVs from vascular and resident cells by facilitating exchange of biological information between neighboring cells serve as cellular effectors in the bloodstream and play a key role in all stages of disease progression. This article reviews the current knowledge on the role of MVs in AT and CVD. Attention is focused on novel aspects of MV-mediated regulatory mechanisms from endothelial dysfunction, vascular wall inflammation, oxidative stress, and apoptosis to coagulation and thrombosis in the progression and development of atherothrombosis. MV contribution to vascular remodeling is also discussed, with a particular emphasis on the effect of MVs on the crosstalk between endothelial cells and smooth muscle cells, and their role regulating the active process of AT-driven angiogenesis and neovascularization. This review also highlights the latest findings and main challenges on the potential prognostic, diagnostic, and therapeutic value of cell-derived MVs in CVD. In summary, MVs have emerged as new regulators of biological functions in atherothrombosis and might be instrumental in cardiovascular precision medicine; however, significant efforts are still needed to translate into clinics the latest findings on MV regulation and function.

## Introduction

Despite significant advances in prevention, diagnosis, and therapeutic intervention focused on strategies for preventing cardiovascular disease (CVD), coronary artery disease (CAD) remains the leading cause of mortality and morbidity worldwide. Since not every individual has the same risk of developing future cardiovascular events (CVEs), an important challenge in cardiovascular medicine is to accurately predict who will develop atherosclerosis (AT)-related complications, such as acute coronary syndromes (ACS). Current tools to predict atherosclerotic vascular complications perform only poor to moderate. This emphasizes that there is an unmet need for novel biomarkers to stratify the risk of atherosclerotic complications in the intermediate and high-risk population on top of classical risk factors.

Atherosclerosis, the hallmark sign of CVD, is a silent hypercholesterolemia-triggered chronic systemic inflammatory process of the artery wall that is characterized by deposition of lipids within the intima of elastic arteries. This leads to structural damage and formation of fatty streaks with subsequent loss of elasticity, which can develop to fiber and atheromatous plaques, resulting in thrombus formation and narrowing of the luminal. Damage to arterial endothelial cells (ECs) is considered the earliest event in atherogenesis. Development of atherosclerotic plaques also includes the continuous crosstalk between EC, smooth muscle cells (SMCs), inflammatory cells, and inflammatory mediators (1) acting through mechanisms that have not yet been completely revealed.

**Figure 1:**
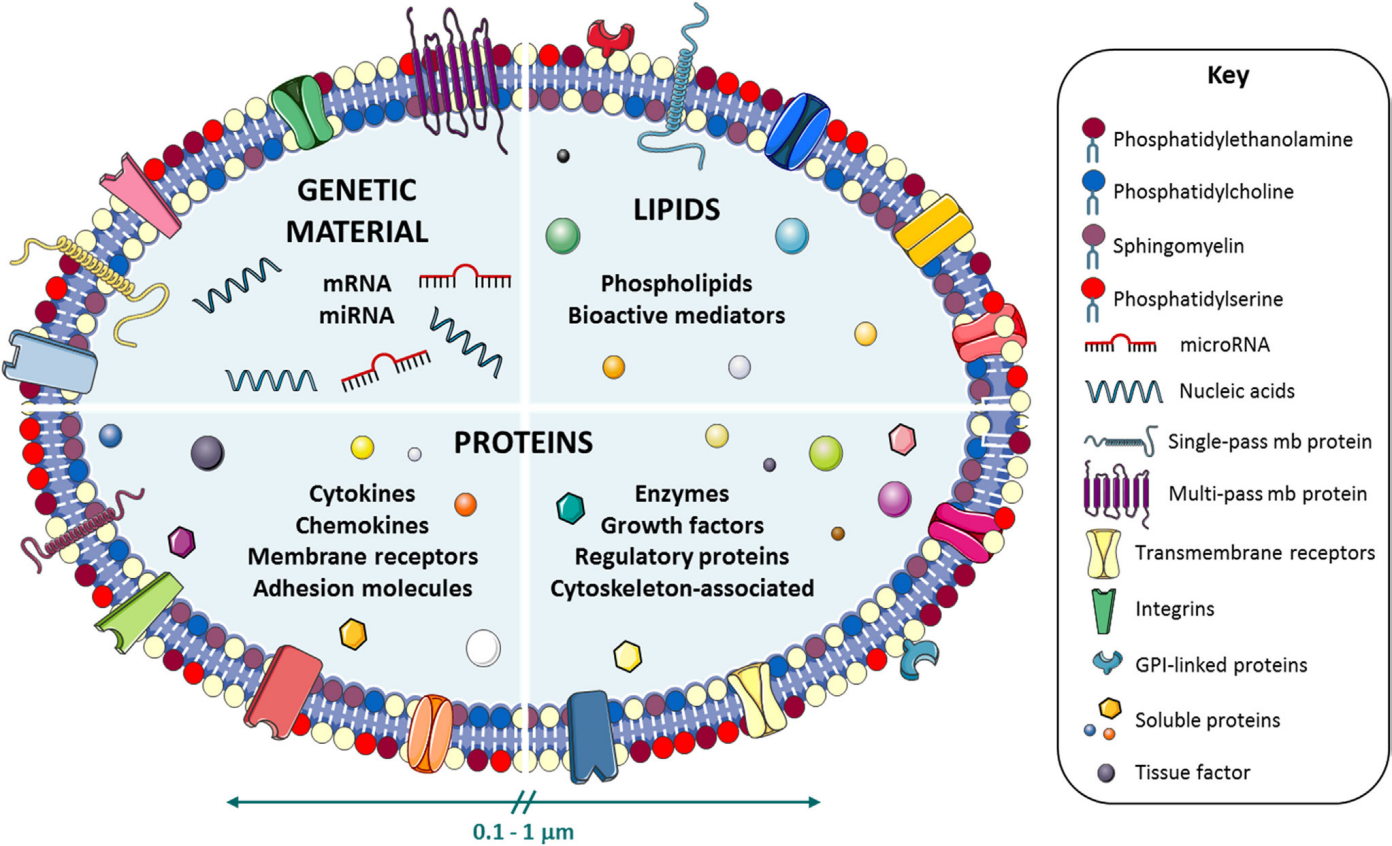
Microvesicle (MV) composition. Schematic representation of the molecular repertoire of the cell-derived MVs. MVs are loaded with distinct components of genetic material [nucleic acids, mRNAs, microRNAs (miRNAs)], lipids (phospholipids and bioactive mediators), and proteins (cytokines, chemokines, membrane receptors, adhesion molecules, enzymes, growth factors, and cytoskeleton-associated and regulatory proteins) to mediate intercellular communication processes.

Microvesicles (MVs), with a diameter ranging from 0.10 to 1.00 μm, are small plasma membrane vesicle fragments, also known as microparticles (MPs), that are released by many cell types, such as platelets, leukocytes, ECs, erythrocytes, and SMCs, into different bodily fluids, including plasma, urine, saliva, milk, sweat, semen, and tears, as well as in conditioned media from cell culture experiments (2, 3) (Figure 1). Depending on the generation mechanism, there are distinct types of extracellular vesicles, including *MVs, apoptotic bodies*, and *exosomes* (4), being MVs the most heterogeneous and studied population so far. The present review particularly focuses on MVs, which are specifically formed by budding of the plasma membrane, a releasing process that is driven by calcium-dependent signaling, activity of several enzymes, cytoskeleton remodeling, and externalization of phosphatidylserine (PS). MVs are shed under basal conditions and their release increases with various stimuli and pathological settings. In contrast to MVs, *apoptotic bodies* are larger permeable membrane vesicles with a diameter >1 μm containing apoptotic nuclear material while *exosomes* constitute the smallest extracellular vesicle type (ranging from 40 to 100 nm in diameter), highly enriched in lipids and tetraspanins, and actively shed from intracellular multivesicular bodies upon fusion with the cell membrane.

Microvesicles are specifically composed of lipids, genetic material, such as mRNA, non-coding ribonucleic acids (RNAs) [microRNA (miRNA)], or even small amounts of DNA, and proteins such as transcription factors, cytokines, and growth factors (Figure 1). Interestingly, the packaging of distinct biomolecules into MVs seems to occur in a non-randomly fashion. Thus, specific miRNAs were seen to be preferentially sorted into MVs. Blood cells and cultured monocytic THP1 cells actively and selectively secreted MV-loaded miRNAs into the circulation in response to various stimuli (5). Nevertheless, further efforts are needed toward a complete understanding of this regulated sorting mechanism. MVs have been characteristically recognized by the externalization of PS on the outer membrane leaflet. However, this property has recently been a matter of debate. New evidence suggests that some MVs can express cell markers without annexin V binding (6, 7). Interestingly, MVs harbor on their surface transmembrane and receptor proteins from the parental cells from which they derived from. This property, important for specific cell–cell interactions, is also used in MV identification and characterization by high-sensitivity flow cytometry. MVs can deliver their cargo to cells nearby or in remote locations, perpetuating the intercellular communication process. Since their content fluctuates depending on the pathological context, MVs have drawn the attention as a potential source of biomarkers for disease identification (8).

Flow cytometry has been the gold standard methodological choice for MV measurements. Recently, some new methods (9) such as atomic force microscopy have been developed. Today there is still a general need of establishing preanalytical steps for MV isolation and of validating novel techniques. Recent efforts (10–12) are addressed to standardize MV analytical procedures between instruments and laboratories (13).

Microvesicles promote the development and progression of AT, by inducing endothelial dysfunction (ED) and initial lesion formation, influencing cell communication, promoting inflammatory reactions and participation in lipid deposition, neovascularization, calcification and unstable plaque progression, and injured plaque clotting and thrombosis after rupture. Here, we review the current and last data on the role of MVs in AT and CVD, highlighting their relevance for vascular remodeling and neovascularization. In addition, we discuss the emerging interest of MVs as prognostic and diagnostic biomarkers of disease and their potential use as therapeutic agents.

## MV-Mediated Regulatory Mechanisms in the Development and Progression of AT

Several evidences support a direct functional role for circulating MVs in atherothrombosis. They span from the early stages related to the presence of classical cardiovascular risk factors (CVRF) (e.g., hyperlipidemia, diabetes, and hypertension) to acute cardiovascular and cerebrovascular events as result of their effect on intercellular communication processes transferring proteins, non-coding RNAs, and even mRNAs (14) to target cells. Specifically, MVs are able to (1) fuse with plasma membrane of target cells and transfer their cargo into the recipient cells, (2) interact with target cells *via* a receptor-mediated signaling mechanism, and (3) be internalized *via* endocytosis and fused with either endosomes to release their content into the cytosol or lysosomes to be degraded (2). To bridge the gap between atherosclerotic lesion initiation, plaque rupture, and CVEs, multifaceted MV-driven molecular mechanisms of AT progression linked to endothelial homeostasis, inflammation, and thrombosis will be discussed.

### Endothelial Dysfunction

Endothelial cells exert natural barrier functions in the vessel wall to prevent pathogen invasion and to maintain vascular integrity, through the balanced release of cell and molecular components acting at paracrine and autocrine level. Vascular ECs change their phenotype and function in response to mechanical injury and systemic factors, such as dyslipidemia, smoking, and other stimuli and/or risk factors.

**Figure 2:**
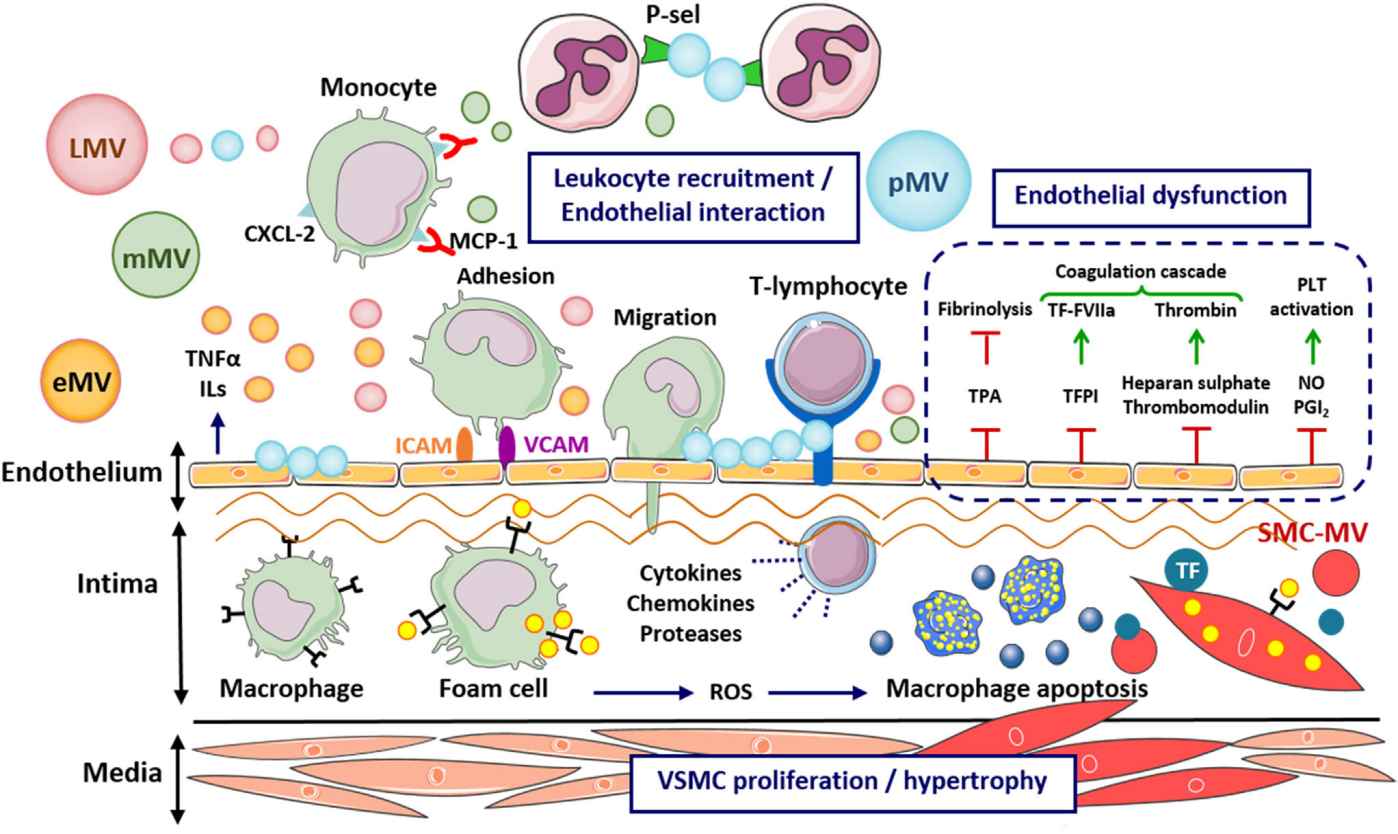
Effects of microvesicles (MVs) on the early stages of atherosclerosis development. Cell-derived MVs are able to interact with the subendothelial matrix, induce endothelial dysfunction, stimulate proinflammatory response, enhance the adhesion and infiltration of leukocytes, as well as oxidative stress, apoptosis, and vascular remodeling, promoting the inflammation and injury of the vessel wall and the progression of atherosclerotic lesions. CXCL-2, C–X–C motif chemokine ligand 2; eMV, endothelial cell-derived microvesicle; FVIIa, coagulation factor VIIa; ICAM, intercellular cell adhesion molecule; LMV, leukocyte-derived microvesicle; mMV, monocyte-derived microvesicle; MCP-1, monocyte chemotactic protein 1; NO, nitric oxide; PGI, prostacyclin; pMV, platelet-derived microvesicle; P-Sel, P-selectin; ROS, reactive oxygen species; SMC-MV, smooth muscle cell-derived microvesicle; VCAM, vascular cell adhesion molecule; TF, tissue factor; TFPI, tissue factor pathway inhibitor; TPA, tissue plasminogen activator; TNF, tumor necrosis factor.

Recent studies support a direct relationship between the levels of circulating endothelial-derived MVs (eMVs) and the degree of ED in patients with end-stage renal failure (15), congenital heart disease (16), CAD (17, 18), type 2 diabetes (T2D) (19), and obesity and hypertension (20). Indeed, MVs are able to interact with the vascular endothelium and promote cellular dysfunction (Figure 2). Thus, MVs from T2D patients have shown to attenuate eNOS expression in cultured EC (21). Furthermore, MVs of endothelial origin have shown to decrease nitric oxide (NO) production *in vitro* (22, 23) and under high glucose conditions through changes in oxidative stress (24) or inducing a temporal cross talk between mitochondria and endoplasmic reticulum (25). These effects have been obtained in *ex vivo* studies (23, 26) and with patient-derived MVs in pathologies such as myocardial infarction (MI) (27), end-stage renal failure (15), metabolic syndrome (28), valvular heart disease and cardiac injury (29), and undergoing percutaneous coronary intervention (30). As a result, vascular tone and endothelial repair capacity are altered. Studies *in vitro* using rat aortic rings provided evidence that eMVs lead to impaired acetylcholine-mediated vasorelaxation (22), and studies *ex vivo* with eMVs from ACS patients induced premature ED, senescence, and thrombogenicity through an angiotensin II-dependent redox activation of phosphoinositide 3-kinase/Akt and mitogen-activated protein kinases pathways (31). In addition, when MVs obtained from plasma of subjects with metabolic syndrome, were injected intravenously in mice, a severe impairment of endothelium-triggered vasorelaxation with decreased NO-synthase expression was detected (28). Horn et al. reported that MVs carry functionally active NO synthase that induce NO production within the MVs, a function downregulated in MVs from patients with ED (32). These results indicate that eMVs have a functional role in the control of vascular homeostasis.

The physiopathological conditions stimulating MVs formation have an impact on MV phenotype and functional activity. Interestingly, Mahmoud and colleagues reported that MVs from healthy individuals or shed under basal conditions had no effects on endothelial function, while *in vitro* generated eMVs exert a protective role on endothelial function in a free-fatty acid-induced model, *via* eNOS/Akt signaling and reduced oxidative stress (33). This recent study reflects on how MVs may behave as different biological effectors in health and disease, highlighting the relevance of the environment and pathological stimuli triggering MV release on their phenotype and biological activity.

Injured endothelium beyond promoting the recruitment of activated platelets to repair damaged ECs, also facilitates adherence of platelet-derived MVs (pMVs) to the vascular wall (Figure 2), inducing in turn the permeability and apoptosis of ECs, the latter by MV transfer of caspase-3 and Rho-kinase enzymes according to the *in vitro* findings reported by Edrissi et al. (34). More specifically, these authors have proved that plasma-derived MVs from rats submitted to chronic cerebral ischemia contain factors involved in the activation of the tumor necrosis factor (TNF)-α pathway that delivered to ECs regulate endothelial permeability *in vitro*.

### Vascular Wall Atheroinflammation

The role of MVs on the propagation of endothelial proinflammatory response was initially reported in *in vitro* studies showing that blood-derived MVs (obtained from either plasma and/or cell cultures, and also induced by high-shear stress) raise the release of cytokines and cell adhesion molecules in ECs (35, 36) and in leukocytes (37). In addition, circulating MVs obtained from healthy volunteers after infusion of a chemotactic peptide induced cytokine and chemokine release in *in vitro* EC cultures (38). Similar findings have been reported using MVs from both animal models and patients (39, 40). Thus, MVs obtained from a high-fat diet-fed obese murine model of insulin resistance and T2D induced the expression of vascular cell adhesion molecule-1 and reactive oxygen species (ROS) production in rat cardiac ECs (41). Furthermore, MVs have also shown to increase the monocyte expression of cell surface antigens such as CD11a/CD18 and CD11b/CD18 α_M_/β_2_ (37) and the endothelial expression of intercellular cell adhesion molecule-1 (ICAM-1) (42, 43). Increasing evidences suggest that regulation of protein expression by MVs is dependent on their miRNA-cargo. Thus, MV-mediated regulation of ICAM-1 expression in ECs is dependent on their content in miRNA-222 and its transfer to the target cells (44). MV-associated inflammatory cascade promotes the binding of monocytes to the endothelium and their infiltration into the atherosclerotic plaque (Figure 2) (24, 37, 45–47). Indeed, *in vitro* studies demonstrated that monocyte and leukocyte activation and adhesion is regulated by eMVs transferring miRNA-10 to the monocyte and thereby targeting nuclear factor-κB inflammatory pathway (48) and by MVs that carry arachidonic acid as well as oxidize phospholipids (45). Another smart study showed that pMVs transfer proatherogenic C–C motif chemokine 5 [regulated on activation, normal T cell expressed and secreted (RANTES)] to either activated ECs or murine atherosclerotic carotid arteries enabling monocyte adherence (46). Thus, MVs might contribute to the early atherosclerotic lesions development by promoting leukocyte adhesion to the endothelium.

### Oxidative Stress, Apoptosis, and Vascular Remodeling

**Figure 3:**
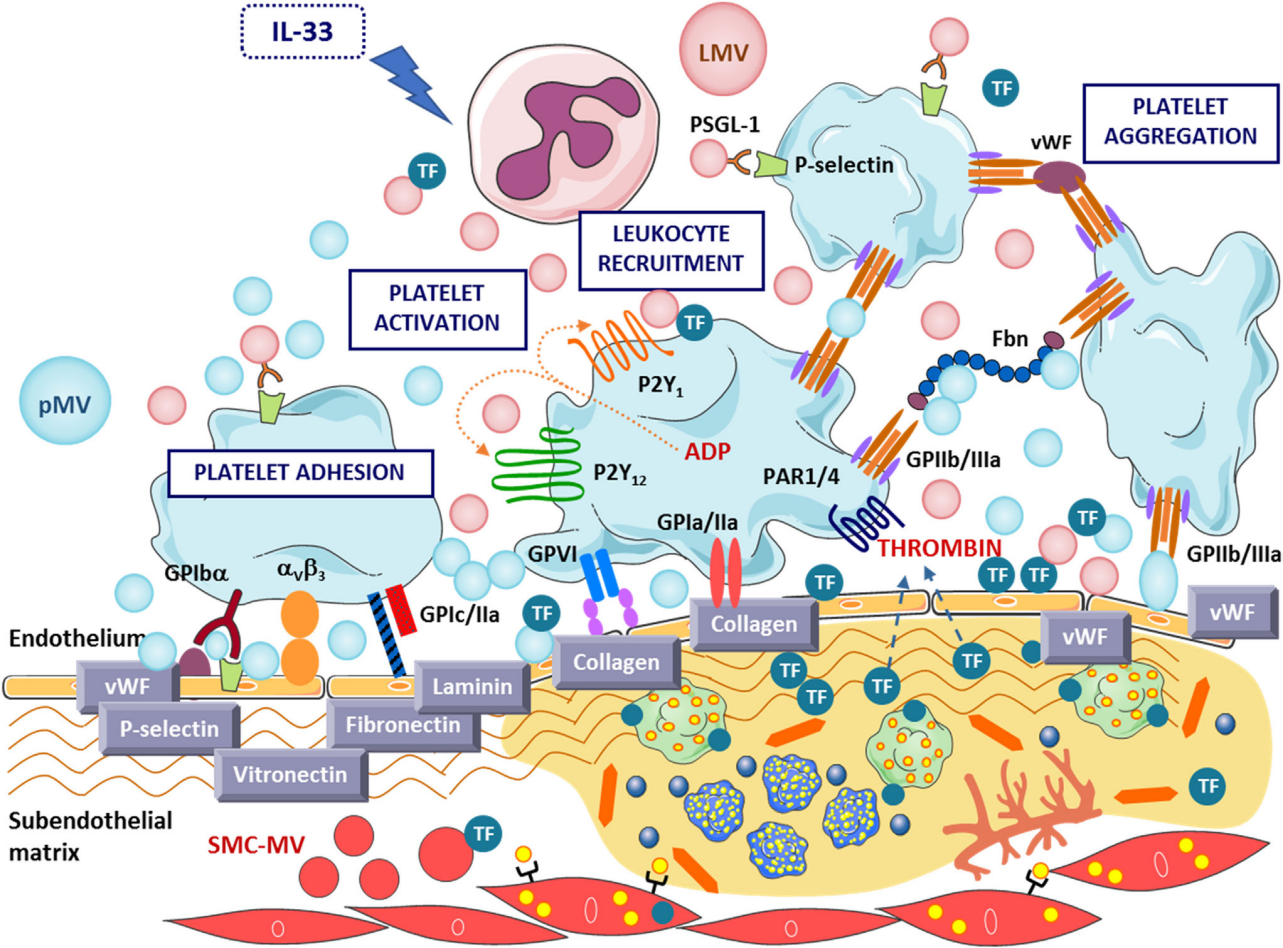
Key prothrombotic mechanisms exerted by microvesicles (MVs) at sites of vascular injury. MVs’ adhesion to the endothelial layer enhances platelet adhesion and thrombus formation and contributes to TF release at the injured atherosclerotic plaque. IL-33, interleukin-33; LMV, leukocyte-derived microvesicle; mMV, monocyte-derived microvesicle; pMV, platelet-derived microvesicle; PSGL-1, P-selectin glycoprotein ligand-1; SMC-MV, smooth muscle cell-derived microvesicle; TF, tissue factor; vWF, von Willebrand factor.

The seminal study of Forlow et al. showed that pMVs expressing P-selectin (P-Sel) promoted monocyte infiltration by facilitating leukocyte–leukocyte interactions under detrimental flow conditions (Figure 2) (49). Besides, lipid-containing MVs derived from various cell types promote foam cell formation and programmed cell death by inducing lipid and cholesterol accumulation in macrophages through a toll-like receptor induced mechanism *in vitro* and in a human skin model (50). TNF-α-induced eMVs contributed to apoptosis and inflammation of ECs *in vitro* (51). Delivery of the proapoptotic enzymes caspase-1 and caspase-3 to target cells by eMVs, pMVs, monocyte-derived MVs (mMVs), T-lymphocyte-derived MVs (ℓMVs), and erythrocyte-derived MVs trigger macrophage cell apoptosis *in vitro* (52–56), which in turn causes more MV release, amplifying the process and facilitating progression of the atherosclerotic lesion. Thus, human atherosclerotic lesions contain MVs (57, 58) being those of monocyte and macrophage origin the most abundant. Apoptosis cell-derived MVs amplify the initiation and maintenance of inflammation in human monocytes together with interferon γ (59) in contrast to a recent study pointing out that MVs released from apoptotic cells provoke less inflammatory response than MVs from viable cells in monocytes, relating this effect with the specific miRNAs pattern found in these MVs (60). Furthermore, atherogenic low-density lipoprotein (LDL) modified by aggregation induce the release MVs enriched in tissue factor (TF) from SMCs (61) (Figures 2 and 3). pMVs increased SMC proliferation (62) by a mechanism independent of platelet-derived growth factor (PDGF) (63) whereas mMVs induced SMC death by caspase-1 (56).

High-risk plaques have a large lipid content covered by a thin fibrous cap penetrated by proinflammatory cells and a diffuse pattern of calcification (64, 65). The level of calcification has associated either with plaque burden (66) and lesion destabilization (67) or with less prone to rupture plaques (68). It seems plausible that the effect of calcification on atherosclerotic lesions evolves from a destabilizing effect in early lesions to a potential stabilizing effect of larger calcium burden in more advanced plaques (69). MVs from ECs, VSMCs, and macrophages seem to be recruited at the site of atherosclerotic plaque calcification and have been associated with the calcification process (70–73). Regarding MV-associated modulation of fibrous cap weakening, several cell-derived MVs can influence the progressive depletion of VSMCs and extracellular matrix degradation through metalloprotease interaction. Specifically, the involved molecular effectors are matrix metalloproteinase 9 (MMP-9), a disintegrin and metalloprotease-10 (ADAM-10) and -17 (ADAM-17) in neutrophil-derived MVs [(74, 75), respectively], MMP-9, matrix metalloproteinase-2 (MMP-2), and MMP-10 in eMVs [(76–78), respectively], and the metalloprotease TNF-α converting enzyme (TACE/ADAM-17) of human atherosclerotic plaques-derived MVs (79). A relevant finding is that the shedding of matrix MVs from arterial intimal SMCs was found higher in athero-prone areas of the human aorta than in athero-resistant areas at the preatherosclerotic disease stage with quantitative electron microscopic analyses (80). Shear stress-induced eMVs-containing miRNA-143 and miRNA-145 prevented VSMC dedifferentiation (81). Likewise, circulating MVs bearing miRNA-223 could penetrate the vascular wall and inhibit VSMC proliferation and migration, resulting in a decreased plaque size (82).

### Coagulation and Thrombosis

The potential role of MVs in atherothrombosis is supported by a large number of studies. Procoagulant MVs are located within human advance vulnerable plaques (83). Upon atherosclerotic plaque erosion or rupture, the high-risk vulnerable plaque exposes their vascular contents to the blood flow, the coagulation cascade is activated and, concomitantly, there is recruitment and activation of circulating platelets that may lead to thrombus formation. Thrombosis may compromise arterial blood flow supply leading to the presentation of oxygen deficiency and the presence of MI.

First described as platelet dust (84), MVs are procoagulant biological effectors due to the surface content of negatively charged PS, which confers them high binding capacity for coagulating factors, being prominently higher in MVs than cell surface membrane in the case of platelets (85). The presence of other molecules and receptors including TF (83), factor VIII and Va (86), P-selectin glycoprotein ligand-1 (PSGL-1) (87), glycoprotein IIb/IIIa (88), and protein disulfide isomerase (PDI) (89) on MV surface might further enhance clot formation and thrombosis. TF can be functionally transferred *via* MVs to monocytes and other cells (90, 91). Protease-activated receptor 2 activation favors the shedding of TF-rich MVs through a process involving filamin A. Specifically, the interaction of TF with filamin A translocate cell surface TF to cholesterol-rich lipid rafts, promoting its activity and release into MVs (92). Indeed, procoagulant TF-rich MVs are increased by hyperinsulinemia (93) and found increased in patients with T2D (94). Recently, it has been shown that proinflammatory cytokine IL-33 induces differential TF expression and activity in intermediate monocyte subset as well as the release of procoagulant MVs rich in TF (95), emphasizing the interplay between inflammation and thrombosis (Figure 3).

Several key studies have addressed how MVs affect the clotting process (Figure 3). Upon endothelial injury, pMVs can bind to subcellular matrix to enhance clotting (96). Noteworthy, we reported that circulating and platelet-derived MVs exert direct effects on atherothrombosis by promoting platelet and fibrin deposition on atherosclerotic arterial wall (97). We performed a proof-of-concept study by perfusing blood with and without exogenously added pMVs to injured atherosclerotic vessel wall and demonstrated that elevated levels of pMVs were able to enhance platelet and fibrin adhesion under conditions of high-shear stress (97, 98). Indeed, a decreased level of pMVs bearing surface epitopes of adhesion and activation was found in blood perfusing prothrombotic surfaces, and also in ST-segment elevation myocardial infarction (STEMI) patients (99), supporting the high tendency to adhere of pMVs (49). High-shear stress-induced pMVs induce enhanced expression of cell adhesion molecules in ECs and monocytes (36) and regulate monocytes involvement in AT and inflammation (37). Interestingly, MVs from healthy subjects support low-grade thrombin generation (100) and are able to activate a stress signaling pathway in ECs leading to increased procoagulant activity (35); and PDI-bearing pMVs promote platelet hyperaggregability and insulin degradation in patients with T2D (89). Furthermore, interactions between platelet P-selection with PSGL-1 of leukocyte-derived MVs (LMVs) are required to concentrate TF activity at the thrombus edge to promote thrombus formation (87). MVs carrying functional TF might enable the growth of unregulated thrombus generation and fibrin formation and thrombus propagation ultimately leading to thrombotic complications (101).

## Potential Prognostic, Diagnostic, and Therapeutic Value of Cell-Derived MVs in AT

CV diseases reflect a continuum of mechanisms underlying the gradually progressing AT. In addition to the pathogenic effects of MVs in AT and thrombosis, circulating levels of MVs (cMVs) of different cellular origin are increased in CVDs and reflect the severity of the different stages of the pathophysiology (102), thus cMVs might serve as potential diagnosis and prognosis biomarkers, which would be valuable tools for cardiovascular risk prediction as well as for evaluating the pharmacological response to therapeutic interventions.

Circulating microvesicles exist in the blood of normal healthy individuals released upon activation and in some cases apoptosis of vascular cells. Indeed, MV levels show gender-specific differences (103, 104) and changes are observed with age (105), during pregnancy (106), after exercise (107–109) and after a high-fat meal (110). It is important to recognize that differences may arise depending on age, gender, body mass index, lipid, hormone levels, smoking status and other confounding variables in apparently healthy subjects when evaluating cMVs for pathogenic potential (111). Another relevant issue to bear in mind when studying cMVs is their clearance mechanism, which might influence the levels of cMVs and could also be impaired by CVRF.

### Cardiovascular Risk Factors

The number and phenotype of cMVs has been associated to major CVRF, such as smoking, diabetes mellitus, obesity, hypertension, dyslipidemia, and metabolic syndrome (Table 1). Within hyperlipidemia, it has been shown that in patients with heterozygous familial hypercholesterolemia (FH), pMVs carrying TF identify subclinical atherosclerotic plaque burden (112), and ℓMPs are able to discriminate between lipid-rich and fibrous atherosclerotic plaques in the same patients (113), thus reflecting that chronic exposure to high levels of LDL activates the vascular compartment by distinct mechanisms. Therefore, CVRF correlate with increased numbers of MVs indicating that MVs might be active in triggering thrombosis and further contributing to an increased risk of CVEs.

**Table 1:** Microvesicle-associated prognostic and diagnostic value in cardiovascular risk stratification and CVDs.

CVRF and atherothrombotic diseases		Reference
CVRF	Smoking	(114–116, 117)

	DM	Insulin resistance (118) DM (119–123) Type 2 DM (124–127) DM with microvascular complications (128)

	Obesity	In children (129) In adults (114, 130, 131) Caloric restriction/bariatric surgery (132)

	Hypertension	(18, 133–135)

	Dyslipidemia	Dyslipidemia (112, 113, 126, 136)

	Metabolic syndrome	Clustering of CVRF (28, 137–139) Cardiometabolic risk (140)

	Uremia	(141)

Atherosclerosis (AT)	FRS	(140, 142)

	ED	(15, 115)

	Calcification	(143)

	PAD	(105, 144)

	Subclinical AT	(19, 112, 113, 145)

	Stable CAD	(17, 146–148)

CVD	ACS	(142, 146, 149–154) STEMI (155, 156)

	Cerebrovascular disease	Ischemic stroke (157–162) Cerebral vasospasm (163) Lacunar infarcts (157) Carotid AT (164)

*ACS, acute coronary syndromes; CAD, coronary artery disease; CVD, cardiovascular disease; CVRF, cardiovascular risk factors; DM, diabetes mellitus; ED, endothelial dysfunction; FRS, Framingham Risk Score; PAD, peripheral artery disease; STEMI, ST-segment elevation myocardial infarction*.

### Atherosclerosis

Several studies have shown an association between cMVs and the Framingham Risk Score (used to predict CVD risk), ED, coronary calcification, peripheral artery disease, and subclinical AT (Table 1). Our group has recently reported that subjects at high cardiovascular risk who were about to develop a major CVE have increased levels of blood CD3^+^/CD45^+^ cMV (165). This is in agreement with our previous findings demonstrating that FH patients with subclinical lipid-rich atherosclerotic plaques have significantly higher levels blood-derived circulating ℓMVs than those with fibrous plaques (113). Besides, levels of circulating MVs have been found increased in patients with stable CAD, being MVs of endothelial origin (CD144^+^, CD31^+^/Annexin V^+^) and bearing miRNA-199a and miRNA-126 the most associated to major adverse CVEs (17, 146–148).

### Acute Coronary Syndromes

Cell-derived cMV levels are profoundly increased in patients with ACS (Table 1). In a study in patients with STEMI, our group has demonstrated increased levels of pan-LMPs (CD45^+^), including lymphocytes (CD3^+^) and monocytes (CD14^+^) within the first hours of the ACS onset, with partial reductions after 72 h, likely because the inflammatory burst occurred at STEMI onset (155). These data are strongly supported by the fact that platelet- and monocyte-derived cMVs also associate with AMI severity (156) and CVD mortality (166), reflecting the sustained underlying endothelial injury and leukocyte and platelet activation in CVD progression after a CVE.

### Cerebrovascular Disease

Circulating microvesicles have been associated with ischemic stroke, cerebral vasospasm, lacunar infarcts and multi-infarct dementia, and carotid AT (Table 1). Besides, a recent case-control pilot study suggests that circulating MVs derived from monocytes of M2 phenotype, but not those of M1-phenotype are significantly increased in patients with intracerebral hemorrhage within 12 h of symptom onset (167).

## AT-Driven Angiogenesis and Neovascularization: MVs as Therapeutic Vectors

Angiogenesis is an active fine-tuning process of vessel sprouting and growth that depends on a precise interplay between stimulatory and inhibitory signals of ECs, SMCs, and pericytes (168). Notably, recent evidence outlines the importance of EC metabolism for angiogenesis in the context of atherogenesis and AT progression (169). Thus, angiogenic processes have severe consequences on vascular remodeling and plaque stability within vulnerable atherosclerotic plaques (plaque rupture and intra-plaque hemorrhage). The generation of new delicate and frail vessels within the growing atherosclerotic lesion contributes to increase the vulnerability of the plaque to rupture. Intriguingly, it is not fully understood whether an atherosclerotic plaque in the arterial tree will rupture and trigger thrombotic cascade or will stabilize. The highest accumulation of neovessels was found in ACS-related human coronary plaques (170). Paracrine intercellular communication exerted by MVs might play a role in the adaptive response of ischemic tissue to hypoxic stress caused by CVD (171). Interestingly, MVs have been postulated as both pro- and antiangiogenic factors (172–177) (Table 2). Indeed, low levels of eMVs-containing β1-integrin and the enzymatically active MMP-2 and MMP-9 showed to promote angiogenesis whereas high levels abolished the angiogenic effects (76). The effect of MVs on angiogenesis is also highly dependent on the quantity, the parental cell type and cell surface content.

**Table 2:** Proangiogenic potential of distinct cell-derived MVs.

Type of MVs	Source	Function	Mechanism	Reference
Platelet-derived MVs	Patients with atherosclerosis	↑ Neovascularization of CAC	Mediated by RANTES	(178)
	Healthy donors	↑ Angiogenesis *in vitro* and *in vivo*	Dependent on VEGF	(172)
	Healthy donors	↑ Proliferation, angiogenesis and neurogenesis	Differentiation and proliferation of stem cells mediated by MV growth factors (FGF2, VEGF, and PDGF)	(179)
	Healthy rats	Protective effect against cerebral ischemic–reperfusion injury	Mediating remote ischemic preconditioning	(180)
	Healthy donors	↑ Tube formation	*Via* the pertussis toxin-sensitive G protein and the PI3K pathway	(181)
	Healthy donors	↑ Capillary tube formation and reendothelialization	By sensitization of CXCR4 and growth factors	(182)

Endothelial cell (EC)-derived MVs	Mice ischemic hindlimb muscle	↑ Postischemic neovascularization	–	(183)
	Human umbilical vein ECs	↑ Angiogenesis (with low levels of eMVs)	Through β1-integrin and MMP-2 and -9	(76)
	Human ECs	↑ Formation of capillary-like structures	By MV-harboring Sonic Hedgehog	(174)
	Human coronary artery ECs	↑ Vascular endothelial repair	Induced by miR-126-containing MVs	(24)
	Human microvascular EC	↑ *In vitro* tube formation	MV-induced plasmin generation	(184)
	Human umbilical vein ECs	↑ Angiogenesis	By upregulating MMP expression	(185)

Carotid plaque-derived MVs	Endarterectomy specimens	↑ *In vivo* the tube formation	In a CD40 ligand-dependent manner	(186)

T-lymphocyte-derived MVs	Human lymphoid cells	↑ Neoangiogenesis	By NO synthesis pathway	(175)

Myofibroblasts-derived MVs	Skin wound myofibroblasts	↑ Capillary formation	–	(187)

EPC-derived MVs	EPCs from healthy donors	↑ Angiogenesis *in vitro* and *in vivo*	Through eNOS and PI3K/Akt pathway	(188)

MSC-derived MVs	MSC from bone marrow	Promote angiogenesis	–	(189)
	MSC from bone marrow	↑ Postischemic angioneurogenesis	–	(190)
	MSC from umbilical cord	↑ Angiogenesis *in vitro* and *in vivo*	–	(191)
	MSC from umbilical cord	↑ Angiogenesis *in vitro* and *in vivo*	By ↑ VEGF in a HIF-1α independent manner	(192)

*CAC, circulating angiogenic cells; CXCR4, C–X–C chemokine receptor type 4; EPC, endothelial progenitor cell; eNOS, endothelial NO synthase; FGF, fibroblast growth factor; HIF, hypoxia-inducible factor; MSC, mesenchymal stem cell; MMP, matrix metalloproteinase; MV, microvesicle; NO, nitric oxide; PDGF, platelet-derived growth factor; PI3K, phosphatidylinositol-3-kinase; RANTES, regulated on activation, normal T cell expressed and secreted; VEGF, vascular endothelial growth factor; MV, microvesicle; MMP-2, matrix metalloproteinase-2*.

Microvesicles from apoptotic ECs (24, 188), endothelial progenitor cells (EPCs) (188), platelets (172, 181, 182, 193, 194), skin wound myofibroblasts (187), and ischemic muscle (183) stimulate endothelial proliferation, survival, migration, repair, and tube formation *in vitro* by activating pro-angiogenic signaling cascades, such as ERK and PI3K/Akt pathways, or through upregulation of MMP-2 and MMP-9 expression in ECs (185) (Table 2). Leroyer et al. (186) elegantly demonstrated that carotid plaque MVs stimulate both endothelial proliferation and *in vivo* angiogenesis in a CD40 ligand-dependent manner, which could be modulated by the fibrinolytic activity of eMVs and LMVs (184). Indeed, eMVs play an important role in plasmin formation, which can influence *in vitro* the tube formation of EPCs in a dose-dependent manner (184).

Other cell-specific MVs have also shown proangiogenic potential (Table 2). pMVs from atherosclerotic patients increased the neovascularization capacity of circulating angiogenic cells through a RANTES-mediated mechanism (178). pMVs not only were shown to stimulate vascular endothelial growth factor (VEGF)-dependent revascularization after chronic cardiac ischemia (172) but also stem cell repair mechanisms after brain ischemia in rats by increasing angiogenesis and neurogenesis at the infarct zone (179). In addition, the remote conditioning protective effect of pMVs was further proved against cerebral ischemic reperfusion injury (180). Increased levels of MVs from ischemic muscle showed to promote postischemic neovascularization in mouse after hindlimb ischemia (HLI) (183). T-lymphocyte-derived ℓMVs enriched with the morphogen sonic hedgehog increased neoangiogenesis and restored endothelial function after injection in mice by stimulating the NO synthesis pathway (175, 195). Endothelial colony-forming cell-derived MVs were demonstrated to be pro-angiogenic both *in vitro* and *in vivo* through eNOS and the PI3K/AKT pathway (188). Moreover, MVs from bone marrow-derived mesenchymal stem cells (MSCs) showed proangiogenic activities in a rat MI model contributing to cardiac repair (189) and improved postischemic neuroangiogenesis in a stroke model (190) while MSC–derived MVs from umbilical cord enhanced tube-like structure development and further rescued blood flow in a rat model of HLI (191). Indeed, umbilical cord MSC-derived MVs were shown to enhance angiogenesis in a rat model of kidney ischemia (192), suggesting that MSC-derived MVs might exert cardioprotective effects.

In contrast to the findings reported above, placental MVs have been implicated in antiangiogenic processes contributing to impaired perfusion of placenta in patients with preeclampsia (196). In line with these studies, high levels of eMVs inhibited angiogenesis in the cultured segments of hearts by hampering endothelial nitric oxide synthase (eNOS) regulation (176). Oxidative stress is also involved in the antiangiogenic effect of MVs. Lymphocyte-MVs inhibited angiogenesis regulating negatively VEGF pathway (197) whereas eMVs inhibited *in vitro* angiogenesis by impairing acetylcholine-induced endothelial vasorelaxation and NO production in rat aortic rings (22, 173). Recently, eMVs were reported to inhibit capillary-like branch structure formation by microvascular ECs (_m_ECs) *in vitro* and *in vivo* triggered by ROS *via* the expression of CD36 on the target EC (198).

Interestingly, the stimulating environment influencing the generation of MVs is able to switch their angiogenic phenotype. Treatment of MSCs with PDGF generated proangiogenic MVs (199), showing that the cargoes of MVs greatly impact on their effects. In agreement, cavin-2 that is released in eMVs and required for eMV biogenesis acts as a regulator of angiogenesis by producing NO and controlling the stability and activity of eNOS (200). Moreover, we found that C-reactive protein (CRP) is pro-angiogenic (201) and interestingly CRP is carried by circulating MVs in ischemic patients (202). eMVs from patients with chronic thromboembolic pulmonary hypertension increased EC endoglin concentration and stimulated endothelial angiogenesis showing a protective mechanism for ED and vascular occlusion (203). Of note, exercise intensity increases the levels of circulating pMVs with proangiogenic potential, which stimulate EC proliferation, migration, and tubule formation (204). Besides, antihypertensive drugs have shown to regulate both eMV generation and angiogenesis (205). Therefore, MVs might be considered as dual-purpose mediators of cell–cell communication in health and disease, greatly depending on the surrounding environment in which they are formed, their content and the pathophysiological context where they exert their functions.

Similarly, MVs from preconditioned adipose-derived stem cells (ASC) showed proangiogenic potential through the release of miR-31 targeting factor-inhibiting HIF-1 and enhancing ASC therapeutic efficacy (206). Indeed, miRNA delivery is a key mechanism involved in the effects of MV-driven angiogenesis such as EPC-MVs promoting neovascularization and impairing muscle damage after HLI (207) or inducing the survival of human islet transplants (208). Thus, eMVs released upon interleukin-3 activation transferred miRNA-126-3p and pSTAT5 into ECs thereby promoting angiogenesis (209). In this regard, MVs containing miRNA-126 stimulate reendothelialization after vascular injury (24) and administration of vesicles containing miR-126 decreased atherosclerotic plaque formation and favored plaque stability in mice (210). Besides, transfer of miRNA-150 to ECs by mMVs also promoted angiogenesis both *in vitro* and *in vivo* (117).

**Figure 4:**
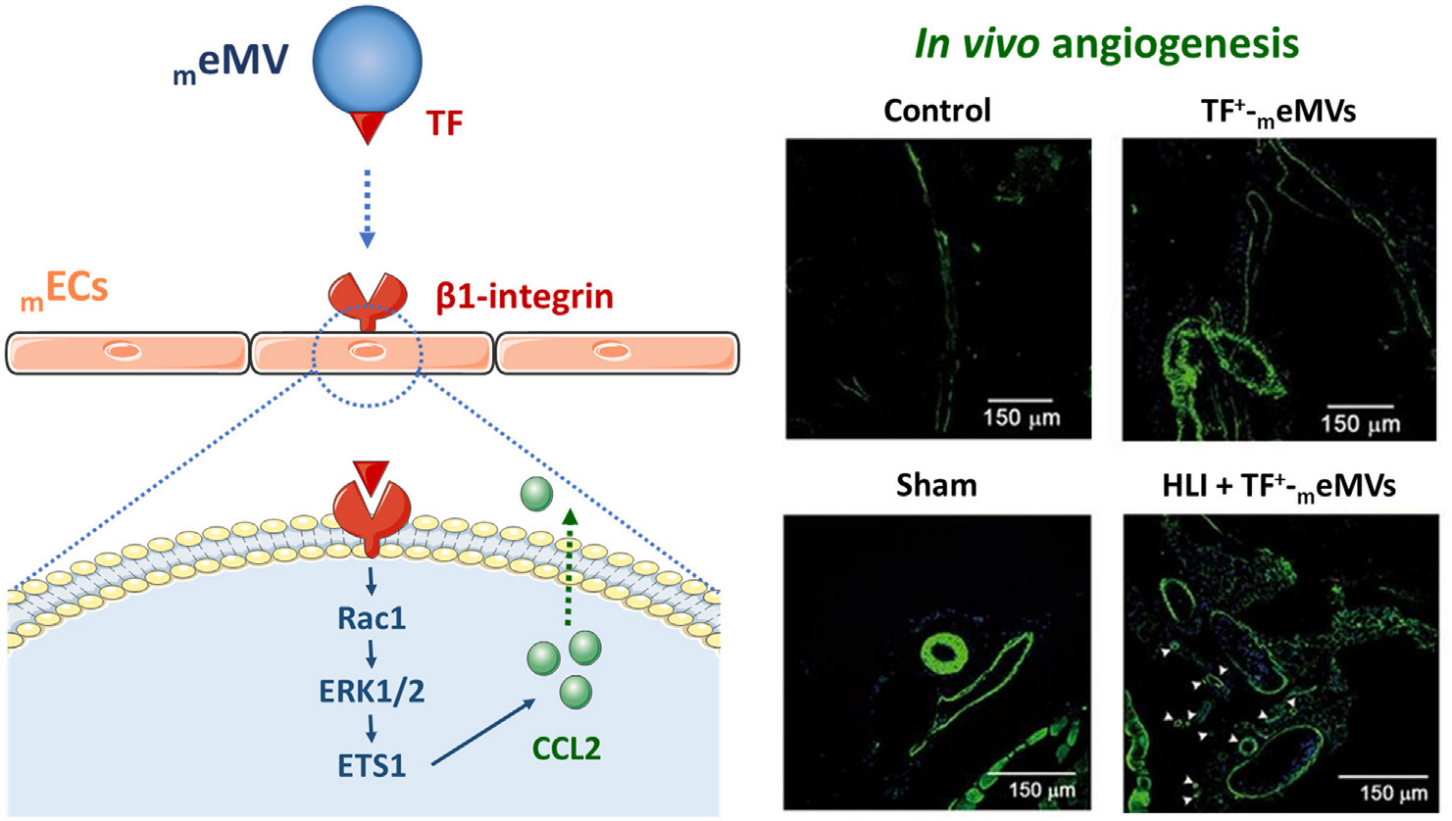
Microvesicle (MV)-mediated neovascularization. TF-positive microvascular endothelial-derived MVs (TF^+^-_m_eMVs) were shown to interact with endothelial cell surface β1-integrin to induce a Rac1–ERK1/2–ETS signaling cascade that leads to CCL2 production and angiogenesis (216). Representative immunofluorescence images demonstrate that TF^+^-_m_eMVs enhance collateral capillary formation and angiogenesis *in vivo* after ischemic hindlimbs femoral arteriectomy with antibody against α-actin (green) and nuclear staining (blue). HLI, hindlimb ischemia; _m_eMV, microvascular endothelial-derived microvesicle; TF, tissue factor.

Tissue factor, the primary cellular initiator of blood coagulation, is also involved in angiogenic processes (211–213). Proangiogenic signaling through TF-dependent MV-mediated activation of PAR-2 has been reported in hypoxic ECs (214). Besides, TF^+^-MVs were shown to bind to β1-integrin in the surface of ECs to induce proliferation through ERK1/2 (215). In the same line, we have demonstrated that TF-containing eMVs from microvascular ECs (_m_eMVs) interacted *via* paracrine signaling with other _m_ECs and triggered angiogenesis *ex vivo* and postischemic collateral vessel growth *in vivo* (216). The _m_eMVs proangiogenic potential was shown to be regulated by β1-integrin–EC interactions inducing Rac1–ERK1/2–ETS signaling and CCL2 production (Figure 4). Taken together, MVs can overwhelm the effects of arterial occlusion and tissue ischemia by stimulating postischemic neovascularization together with tissue reperfusion. Beyond being a promising therapeutic strategy for treating ischemic diseases (217), angiogenesis has also a role in the context of tumor progression and cancer, in which distinct types of MVs by means of cell-cell communication have also a regulatory function (14, 218–228). It is important to join efforts toward the ultimate goal of reaching therapeutical applications of MVs into the clinical arena.

## Pharmacological and Non-Pharmacological Intervention

A deep understanding of the role of MVs in AT might be fundamental for both CVD risk factor control and therapeutic treatment (130). Non-pharmacological interventions lie in overcoming CVRF by lifestyle modifications, such as exercise and diet. In primary prevention, a recent clinical trial showed the benefit of the adherence to the Mediterranean diet in patients at high cardiovascular risk in relation to the incidence of severe CVEs (229). Within the same population, our group has recently demonstrated that decreased levels of cMVs derived from ECs, leukocytes and activated platelets could signal for a reduced rate of major CVEs in high-risk patients under state-of-the-art treatment and receiving a controlled MedDiet supplemented with nuts (230). Dietary modulation of MV release is a relatively new field of study based mainly on short-term studies; further large-scale studies will help to better understand the complex relationship between diet and CVD.

Several drugs have shown to influence cMVs levels (231, 232), their cargoes (233) and even their function (Table 3). For instance, berberine improved endothelial function by reducing CD31^+^/CD42^−^ eMVs and thereby levels of oxidative stress in humans (234, 235) and the adhesion of monocytes to ECs was partially prevented by eMVs after lipid-lowering and antihypertensive treatments (236). Since MV biogenesis and release is not fully understood, distinct therapeutic options are under investigation. Reduced levels of pMVs are associated with the use of distinct anti-platelet drugs such as GPIIb/IIIa inhibitors (237, 238), clopidogrel (239, 240), ticlopidine (121, 122), and acetylsalicylic acid (ASA) (241). In patients under antithrombotic therapy, P-Sel and TF-containing pMVs remain high 6 months after treatment initiation (242), likely due to the fact that low doses of ASA might not be potent enough to prevent the release of pMVs into the microcirculation (243). Our group has reported that ASA intake in patients with diabetes in primary prevention has no effect on pMVs (244). Similarly, antihypertensive drugs like angiotensin II receptor antagonists (245) and calcium channel blockers (246), antioxidants (247), peroxisome proliferator-activator receptor activators (248, 249), hydroxyurea in sickle cell disease (250), and eculizumab in paroxysmal nocturnal hemoglobinuria (251) have also shown influence on MV shedding. Up to now the effect of statins, the cornerstone drugs for lipid-lowering treatment (LLT) in CVD prevention, has been highly debated. While some authors demonstrated that statins may enhance the shedding of MVs (252, 253) many studies have found that statin treatment promote MV inhibition (88, 135, 254–256). Thus, pravastatin and simvastatin decreased pMVs in hypertensive (257) and type-2 diabetic (88, 120) patients. Similarly, atorvastatin diminished the formation of thrombin and the expression of P-Sel, TF, and GPIIIa on pMVs in patients with peripheral artery disease (233) and with dyslipidemia and type-1 diabetes (258). Indeed, in a study focused on the effects of LLT on levels of cMV in atherosclerotic patients in primary prevention, we have reported that the plasma of LLT-treated patients presented lower quantity of MVs and lower content of cell surface activation markers than the plasma from untreated patients with the same blood cholesterol levels (255), indicating a direct benefit of LLT with statins in reducing cell membrane shedding, which may have effects in the beneficial protection against AT characteristic of statins by inhibiting MV generation and the triggering of MV-dependent mechanisms. These data are in agreement to results pointing out the broader use of statins decreasing inflammation and suppressing MV release, an effect that is not shown neither with ezetimibe alone (259) nor with ezetimibe combined with statins (260). Furthermore, several inhibitors of MV shedding such as ROCK inhibitors or calpain, among others, are currently under study. Nevertheless, whether the clinical benefit of these pharmacological strategies is directly related to MV decrease deserves further research.

**Table 3:** Main studies evaluating the effects of pharmacological therapies on circulating microvesicles.

Type of drug	Therapy dose	Subjects (*N*)	MV change	Reference
**Anti-platelet**				

GPIIb/IIIa inhibitors	Abciximab: 250 μg/kg bolus + 12 h 0.125 μg/kg/min Eptifibatide: 180 μg/kg bolus + 18 h 2 μg/kg/min	50 ST-segment elevation patients undergoing percutaneous coronary intervention	↓ GPIV^+^-pMVs ↓ CD11a^+^-LMVs	(238)

Clopidogrel	4 weeks, 75 mg/day	26 patients with stable coronary artery disease (CAD)	= CD51^+^-eMVs ↓ CD42^+^/CD31^+^-pMV	(240)

Probucol and ticlopidine	6 months Probucol: 500 mg/day Ticlopidine: 200 mg/day	23 normolipidemic controls and 53 hyperlipidemic patients	↓ CD62P^+^-pMVs ↓ CD63^+^-pMVs ↓ Annexin V^+^-MVs ↓ CD14^+^-mMVs	(121)

Ticlopidine	1 month, 200 mg/day	21 type-2 diabetic patients	↓ CD62P^+^-pMVs ↓ CD14^+^-mMVs	(122)

Acetylsalicylic acid	8 weeks, 100 mg/day	15 patients with CAD	↓ eMVs ↓ pMVs	(241)

	6 months, bolus of 500 mg and 75 mg/day	51 patients with acute coronary syndromes	↑ CD62P^+^-pMVs ↑ CD142^+^-pMVs	(242)

	10 days, 100 mg/day	43 patients with diabetes	= pMVs	(244)

Cilostazol	1 month, 150 mg/day	30 controls and 43 non-insulin dependent diabetes mellitus	↓ CD62P^+^-pMVs ↓ CD63^+^-pMVs ↓ Annexin V^+^-MVs	(231)

**Antihypertensive**				

Angiotensin II receptor antagonists	Eprosartan: 2 months, 600 mg/day	31 hypertensive and 31 normotensive patients	↓ CD42b^+^-pMVs	(245)

Losartan and simvastatin	24 weeks Losartan: 50 mg/day Simvastatin: 10 mg/day	41 hypertensive patients with hyperlipidemia and/or type-2 diabetes	↓ KMP9^+^-pMVs ↓ CD51^+^-eMVs	(135)

**Anti-diabetic**				

Miglitol	1 month, 150 mg/day	72 non-diabetic patients (37 with hypertension, 35 with hyperlipidemia) and 38 diabetic patients	↓ CD42a/b^+^-pMVs	(232)

Berberine	1 month, 1.2 g/day	14 vs. 11 healthy subjects	↓ CD31^+^/CD42^−^ eMVs	(234)

	1 month, 1.2 g/day	12 vs. 11 healthy subjects	↓ CD31^+^/CD42^−^ eMVs	(235)

**Lipid lowering**				

Statins	Pravastatin: 8 weeks, 40 mg/day	50 patients with type-2 diabetes	↓ CD61^+^-pMVs	(88)

	Atorvastatin: 8 weeks, 80 mg/day	19 patients with peripheral arterial occlusive disease and hypercholesterolemia	↓ CD62P^+^-pMVs ↓ CD142^+^-pMVs ↓ CD41^+^-pMVs	(233)

	Atorvastatin: 80 mg/day	19 patients with peripheral arterial occlusive disease	↓ CD62P^+^-pMVs ↓ CD142^+^-pMVs ↑ CD144^+^-eMVs	(253)

	Simvastatin: 80 mg/day; pravastatin: 40 mg/day; lovastatin: 80 mg/day; fluvastatin: 80 mg/day; atorvastatin: 80 mg/day; rosuvastatin: 20–40 mg/day	37 hypercholesterolaemic patients and 37 normocholesterolaemic controls	↓ CD41^+^/61^+^-pMVs ↓ CD146^+^/31^+^-eMVs ↓ CD45^+^-LMVs ↓ CD14^+^-mMVs ↓ CD142^+^-MVs	(255)

	Simvastatin: 6 months, 20 mg/day	21 hyperlipidemic patients	↓ CD61^+^-pMVs	(256)

	Simvastatin: 24 weeks, 10 mg/day	41 hypertensive patients	↓ KMP9^+^-pMVs	(257)
	Atorvastatin: 2 months, 20 mg/day	20 patients with type 1 diabetes and dyslipidemia	↓ pMVs	(258)

Ezetimibe	10 mg/day	63 patients with coronary heart disease	= MVs	(259)

Ezetimibe with statins	Atorvastatin 80 mg/day vs. atorvastatin 20 mg/day plus ezetimibe 10 mg/day	75 high-risk subjects	↓ pMV with high-dose statin monotherapy	(260)

*MV, microvesicle; eMVs, endothelial-derived microvesicles; LMVs, leukocyte-derived microvesicles; mMVs, monocyte-derived microvesicles; MVs, microvesicles; pMVs, platelet-derived microvesicles*.

### Therapeutic Potential

In addition to pharmacological modulation, the advantageous characteristics of cell-derived MVs, which are a naturally produced therapeutic agents with potential to be used as delivery drugs to specific cell types (261), open up their potential therapeutic application, especially in cardiac cell therapy. Preclinical studies demonstrated that treatment with the vesicular fraction of the conditioned media of hypoxic MSCs decreased infarct size and improved cardiac function by decreasing oxidative stress, enhancing myocardial viability, and preventing damage to the heart after MI in mouse and pig models (262–264). The mechanism of action might involve the transfer of specific RNAs through embryonic stem cell MVs (265). MSC-derived MVs were able to face the detrimental effects of ischemia and reperfusion (I/R) injury in the kidney (266). Similarly, MVs derived from myocardial ischemia could protect hearts from I/R injury in rats through calcium regulatory proteins to alleviate intrinsic myocardial mitochondrial and endoplasmic reticulum apoptotic pathways (267). Interestingly, the effect of cardiosphere-derived cells (CDC) on the therapeutic regeneration of the infarcted myocardium shown in a clinical trial (268) seems to be mediated by CDC-derived extracellular vesicles (269). ECs have also been shown to be atheroprotective by transferring miRNAs *via* MVs to SMCs (81). Specifically, MVs transported functional miR-143/145 into SMCs after activation by shear stress and they reduced atherosclerotic lesion formation in the aorta. Furthermore, a promising therapeutic application of MVs is the use of synthetic MVs mimicking the natural ones. MV delivery could be clinically useful in several conditions such as MI and inflammatory pathologies. Indeed, a recent work reported that infusion of artificially produced MVs can improve inflammation and ameliorate symptoms in different mouse models of MI, multiple sclerosis, and kidney injury (270). Therapeutic innovation of MVs is still in its infancy, and its applicability is hampered by many shortcomings, such as the *in vivo* biodistribution of MVs that depends on their cellular origin, their half-life and the route of administration (271). Further understanding of the biological mechanisms, efficiency and feasibility of these MV-based therapies is warranted.

## Conclusion and Future Perspectives

Microvesicles actively contribute to AT progression and complication due to their implicit role in cell-to-cell communication. cMVs not only play a direct biological role in AT and neoangiogenensis but also might be susceptible targets for pharmacological modulation and emerge as potential prognostic and diagnostic biomarkers of atherothrombotic CVD. However, limitations and technological constraints have precluded the complete understanding of mechanisms of MV formation and pathophysiological relevance. Most functional effects of MVs have been evidenced in *in vitro* studies with a predetermined MV concentration that is not always comparable to the pathophysiological situation while their exact role in the clinical setting is dismissed. Despite cMVs emerge as promising biomarker candidates, clinical studies in larger cohorts are required to clearly delineate their role as diagnostic and prognostic markers of disease. Until now, the lack of standardization in preanalytical phase guidelines, the difficult implementation as routine in the clinical laboratory as well as the high cost of cMV measurements have curbed their full clinical characterization. However, recent and advanced sensitive technologies together with consensus within the scientific community will undoubtedly shed light on the cMV potential as biomarkers. Besides, exploring the cMV specific targeting to a selective tissue, how they are produced *in vivo* and their specific genomic, transcriptomic, metabolomic and proteomic content is essential to design efficient therapeutically strategies involving MVs. In the next coming years, we will witness advances and breakthroughs in the area of MVs that will translate into their use in diagnostic and therapeutic innovation.

## Author Contributions

LB, RS, and TP conceived and coordinated the design of the review and wrote the paper. RS made figures. LB and RS edited the paper. All the authors wrote part of the manuscript, provided critical comments, revised the manuscript, and approved the final version of the manuscript.

## Conflict of Interest Statement

The authors declare that the research was conducted in the absence of any commercial or financial relationships that could be construed as a potential conflict of interest.

## Abbreviations

AT, atherosclerosis; CAD, coronary artery disease; cMV, circulating microvesicle; CVD, cardiovascular disease; CVE, cardiovascular event; EC, endothelial cell; eMV, endothelial-derived microvesicle; EPC, endothelial progenitor cell; ErMV, erythrocyte-derived microvesicle; ICAM, intercellular cell adhesion molecule; I/R, ischemia and reperfusion; LDL, low-density lipoprotein; ℓMV, lymphocyte-derived microvesicle; LMV, leukocyte-derived microvesicle; LLT, lipid-lowering treatment; _m_EC, microvascular endothelial cell; _m_eMV, microvascular endothelial-derived microvesicle; MetDiet, Mediterranean diet; miRNA, microRNA; MMP, matrix metalloproteinase; mMV, monocyte-derived microvesicle; MP, microparticle; MSC, mesenchymal stem cell; MV, microvesicle; NO, nitric oxide; pMV, platelet-derived microvesicle; PS, phosphatidylserine; RNA, ribonucleic acid; ROS, reactive oxygen species; SMC, smooth muscle cell; TF, tissue factor; T2D, type 2 diabetes; VCAM, vascular cell adhesion molecule.
